# Replication Bayes factors from evidence updating

**DOI:** 10.3758/s13428-018-1092-x

**Published:** 2018-08-13

**Authors:** Alexander Ly, Alexander Etz, Maarten Marsman, Eric-Jan Wagenmakers

**Affiliations:** 1 Psychological Methods Department, University of Amsterdam, Postbus 15906, 1001 NK Amsterdam, The Netherlands; 2 Machine Learning Group, Centrum Wiskunde & Informatica, Amsterdam, The Netherlands; 3 Department of Cognitive Sciences, University of California, Irvine, CA USA

**Keywords:** Evidence synthesis, Hypothesis testing, Meta-analysis, Replication

## Abstract

We describe a general method that allows experimenters to quantify the evidence from the data of a direct replication attempt given data already acquired from an original study. These so-called replication Bayes factors are a reconceptualization of the ones introduced by Verhagen and Wagenmakers (*Journal of Experimental Psychology: General, 143*(4), 1457–1475 2014) for the common *t* test. This reconceptualization is computationally simpler and generalizes easily to most common experimental designs for which Bayes factors are available.

The past 5 years have witnessed a dramatic increase in interest for replication studies, largely in response to psychology’s “crisis of confidence” (e.g., Pashler & Wagenmakers, 2012). While this crisis is not unique to the field of psychology by any means, psychologists have been at the forefront of efforts to assess and improve reproducibility in science by way of large-scale replication initiatives, such as the Reproducibility Project: Psychology (Open Science Collaboration, 2015), the *Social Psychology* special issue on replication (Nosek & Lakens, 2014), and the various ManyLabs efforts (Ebersole et al., 2016; Klein et al., 2014). Although the importance of direct replication has been contested by some (for an overview of the most common arguments see Zwaan, Etz, Lucas, & Donnellan, 2017), the increasing prominence of replication studies has prompted researchers to examine the question of how to assess, statistically, the degree to which a replication study succeeds or fails.

A number of complementary questions may arise when evaluating replication studies:

1. Completely ignoring the data of the original study, what is the evidence that the effect is present or absent in the replication attempt? (e.g., Marsman et al., 2017).
2. Taking the data of the original study fully into account, what is the evidence that the effect is present or absent in the replication attempt? (e.g., Verhagen & Wagenmakers, 2014).
3. Pooling the data from the original study and the replication attempt, what is the evidence that the effect is present or absent? (e.g., Scheibehenne, Jamil, & Wagenmakers, 2016).
4. Comparing the data from the original study and the replication attempt, what is the evidence that the effect sizes are similar or dissimilar? (e.g., Bayarri & Mayoral, 2002).

Here we focus on answering the second question using the “replication Bayes factor”, which can be conceptualized as contrasting the position of a hypothetical skeptic and proponent:

“The 1st hypothesis is that of the skeptic and holds that the effect is spurious; this is the null hypothesis that postulates a zero effect size, $\mathcal {H}_{0}: \delta = 0$. The 2nd hypothesis is that of the proponent and holds that the effect is consistent with the one found in the original study, an effect that can be quantified by a posterior distribution. Hence, the 2nd hypothesis—the replication hypothesis—is given by $\mathcal {H}_{r}: \delta \sim $ ‘posterior distribution from original study.’ The weighted-likelihood ratio [i.e., the replication Bayes factor] between $\mathcal {H}_{0}$ and $\mathcal {H}_{r}$ quantifies the evidence that the data provide for replication success and failure.” (Verhagen & Wagenmakers, 2014, p. 1457)

Verhagen and Wagenmakers (2014) proposed this replication Bayes factor in the context of the *t* test, and Wagenmakers et al., (2016b) extended it to the correlation test. The main idea is intuitive: first the original result is summarized by its posterior distribution, and, subsequently, this posterior is used as a prior for the replication attempt. Despite its intuitive appeal in terms of the coherent updating of information, the replication Bayes factor comes with at least three challenges: (1) the procedure is not exact, as the posterior distribution from the original study often needs to be approximated by a convenient function; (2) the procedure requires technicalities and is not easy to apply; (3) the procedure does not generalize well to more complicated designs such as ANOVA (but see George, Ročková, Rosenbaum, Satopää, & Silber, 2017; Harms, 2016; Wagenmakers, Verhagen, & Ly, 2016b).

Here we outline an alternative procedure that solves these challenges. Specifically, the rules of Bayesian updating reveal that the replication Bayes factor quantifies the change in evidence provided by the replication experiment, given that the evidence provided by the original study is already available. This means that any software package that is able to output ordinary Bayes factors can also be used to provide replication Bayes factors, by simply feeding it the combined data set.

Below we first describe the Bayes factor in general terms; subsequently we outline the new conceptualization of the replication Bayes factor and then apply it to a number of concrete examples. We end by discussing the method’s limitations and future challenges.

## The Bayes factor

The Bayes factor is “fundamental to the Bayesian comparison of alternative statistical models” (O’Hagan & Forster, 2004, p. 55) and it represents “the standard Bayesian solution to the hypothesis testing and model selection problems” (Lewis & Raftery, 1997, p. 648) and “the primary tool used in Bayesian inference for hypothesis testing and model selection” (Berger, 2006, p. 378).

Developed and promoted by Jeffreys (1961), the Bayes factor contrasts the predictive performance of two competing models (Etz & Wagenmakers, 1995; Kass & Raftery, 2017; Ly, Verhagen, & Wagenmakers, 2016a, b). Here we focus on the standard scenario that features a null hypothesis, $\mathcal {H}_{0}$, which stipulates the absence of an effect, and an alternative hypothesis, $\mathcal {H}_{1}$, which stipulates the presence of an effect. Both hypotheses are falsifiable in the sense that they make specific predictions about the to-be-observed data. This is accomplished by assigning the model parameters specific values, or—in case the values are unknown and require estimation from the data—entire distributions. For instance, in the case of the *t* test, $\mathcal {H}_{0}$ assigns effect size *δ* in the population a single specific value, namely *δ* = 0 (i.e., the effect is absent); in contrast, $\mathcal {H}_{1}$ assigns effect size *δ* a distribution that reflects the uncertainty about the true effect (e.g., $\delta \sim \mathcal {N}(0,1)$; i.e., the effect is present but likely to be small).

When the competing hypotheses have been adorned with prior distributions, so as to allow concrete predictions about to-be-observed data, the evidence provided by the actually observed data *d* is given by the hypotheses’ relative predictive adequacy for those data (Wagenmakers et al., 2016a):

1 $$ \underbrace{ \frac{P(\mathcal{H}_{1} | d)}{P(\mathcal{H}_{0} | d)}}_{{\text{Posterior model odds}}} = \underbrace{ \frac{p(d | \mathcal{H}_{1})}{p(d | \mathcal{H}_{0})}}_{\begin{array}{cccccccc}\text{Predictive}\\ \text{updating factor}\end{array} } \times \underbrace{ \frac{P(\mathcal{H}_{1})}{P(\mathcal{H}_{0})}}_{{\text{Prior model odds}}} $$

The predictive updating factor—henceforth the Bayes factor—quantifies the change in beliefs about the relative plausibility of the competing hypotheses brought about by the observed data. The prediction that a hypothesis makes for the observed data is obtained by averaging the predictions across the parameter space, weighted by the prior plausibility of the parameter values. For a single hypothesis, this average predictive adequacy is also known as the marginal likelihood or the prior predictive likelihood:

2 $$ \underbrace{ p(d) }_{\begin{array}{ccc}\text{Average}\\ \text{ predictive adequacy} \end{array} } = \overbrace{ {\int}_{\Theta} \underbrace{ f(d | \theta) }_{\begin{array}{cccccccc}\text{likelihood} \\ \text{for a specific } \theta \end{array}} \underbrace{ \pi (\theta) }_{\begin{array}{llll}\text{weighted by the} \\ \text{prior plausibility } \\ \text{of that } \theta. \end{array}}\mathrm{d} \theta}^{\begin{array}{cccccccc}\text{Summed across}\\ \text{all values of } \theta \end{array} }. $$

The Bayes factor is the ratio of the average predictive adequacies for the two competing models:

3 $$ \text{BF}_{10}(d) = \frac{p(d | \mathcal{H}_{1})}{p(d | \mathcal{H}_{0})} = \frac{{\int}_{{\Theta}_{1}} f(d | \theta_{1}, \mathcal{H}_{1}) \pi(\theta_{1} | \mathcal{H}_{1}) \mathrm{d} \theta_{1}} {{\int}_{{\Theta}_{0}} f(d | \theta_{0}, \mathcal{H}_{0}) \pi(\theta_{0} | \mathcal{H}_{0}) \mathrm{d} \theta_{0}}, $$

where *𝜃*_1_ is the parameter vector under $\mathcal {H}_{1}$, and *𝜃*_0_ is the (typically shorter) parameter vector under $ \mathcal {H}_{0}$. Thus, when BF_10_(*d*) = 3, the data *d* are three times more likely under $\mathcal {H}_{1}$ than under $\mathcal {H}_{0}$, and when BF_10_(*d*) = 0.125 (or equivalently, BF_01_(*d*) = 1/BF_10_(*d*) = 8), the data are eight times more likely under $\mathcal {H}_{0}$ than under $\mathcal {H}_{1}$.

The Bayes factor offers several advantages for the analysis of empirical data (e.g., Dienes, 2014; Rouder, 2014; Schönbrodt & Wagenmakers, 2018; Wagenmakers, Marsman, et al., 2018a). Specifically, the Bayes factor allows the researcher to quantify evidence to discriminate between absence of evidence (i.e., BF_01_(*d*) ≈ 1) versus evidence of absence (i.e., BF_01_(*d*) ≫ 1). The Bayes factor also allows one to monitor the evidence as the data come in (Gronau and Wagenmakers, 2017) and to design experiments in order to ensure compelling evidence. Finally, the Bayes factor can also be used to quantify replication success, a topic to which we turn next. For a more detailed introduction to the various fundamental Bayesian concepts, see Wagenmakers et al. (2018a), Wagenmakers et al., (2018b), and Etz and Vandekerckhove (2018).

## Bayesian updating in action

For concreteness, consider the article by Krupenye et al., (2016) titled “Great apes anticipate that other individuals will act according to false beliefs”. In two experiments, the authors used

“(...) an anticipatory looking test (originally developed for human infants) to show that three species of great apes reliably look in anticipation of an agent acting on a location where he falsely believes an object to be, even though the apes themselves know that the object is no longer there. Our results suggest that great apes also operate, at least on an implicit level, with an understanding of false beliefs.” (Krupenye et al., 2016), p. 110.

The Krupenye et al. (2016) article presents two experiments. In each experiment, the apes could either look at the target or at the distractor. Here we start by presenting a Bayesian reanalysis of the first experiment. In this experiment:

“(...) we tested 40 apes [19 chimpanzees, 14 bonobos, and 7 orangutans (...)]. Thirty subjects looked to either the target or the distractor during the central-approach period. Of these 30, 20 looked first at the target (*P* = 0.098, two-tailed binomial test)” (Krupenye et al., 2016, p. 113).

**Fig. 1:**
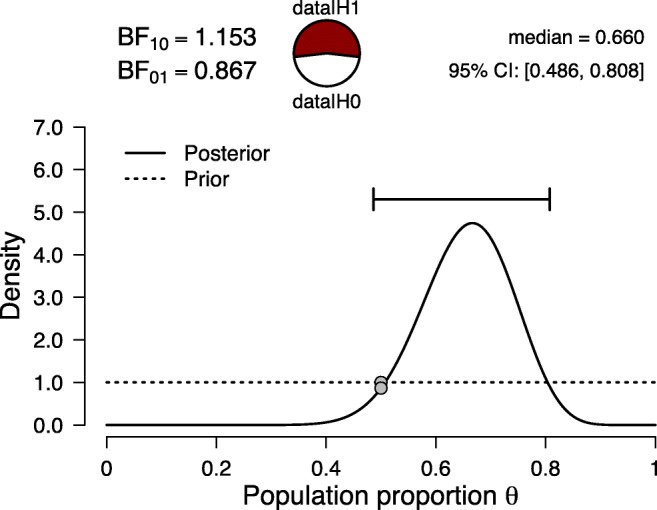
Bayesian reanalysis of the results from the first experiment in Krupenye et al., (2016), where 20 out of 30 apes (≈ 67*%*) first looked at the target. Figure from JASP

Now we reanalyze these results from a Bayesian perspective using the Summary Stats module in JASP (jasp-stats.org; JASP Team, 2018; Ly et al., in press). In our reanalysis, we assume that the data we observe are binomial and governed by a population parameter *𝜃*, the unknown proportion of apes in the population who first look at the target. The hypothesis that the apes are performing at chance level is specified as $ \mathcal {H}_{0}: \theta = 0.5 $. This hypothesis is contrasted with $\mathcal {H}_{1}$, the hypothesis that *𝜃* can take on values other than 0.5. For illustrative purposes, under $\mathcal {H}_{1}$ we assign *𝜃* a default prior distribution of Beta(1,1) that is uniform across the interval from 0 to 1. With the model in place, our uncertainty about the unknown parameter *𝜃* is then updated by the data (i.e., 20 out of 30 looks at the target), and this yields the results shown in Fig. 1.

In Fig. 1, consider the two grey dots that mark the height of the prior and posterior distribution at *𝜃* = 0.5, the null hypothesis of chance performance. These heights can be used to obtain the Savage–Dickey representation of the Bayes factor, an intuitive depiction of its strength and direction: If the dot at *𝜃* = 0.5 gets higher from prior to posterior, the Bayes factor will provide evidence in favor of the null hypothesis (and vice-versa); moreover, the ratio of the heights of the dots exactly equals the Bayes factor (Dickey & Lientz, 1970; Wagenmakers et al., 2010). In this analysis, the two dots are almost at an equal height, and the Bayes factor obtained is BF_10_(*d*) = 1.153, which indicates that the data are non-diagnostic in choosing between the two hypotheses under scrutiny.

We may have gained hardly any evidence for the one hypothesis over the other. However, assume we know that the null hypothesis is false, uninteresting, or generally unworthy of attention. Then we are left with $\mathcal {H}_{1}$, and the corresponding posterior information about *𝜃* is shown as the full curve in Fig. 1. The area under this curve to the right of *𝜃* = 0.5 is much larger than the area to the left of *𝜃* = 0.5; consequently, if we discard the null hypothesis that the apes are performing at chance, thus, only take $\mathcal {H}_{1}$ into consideration, the previously non-diagnostic data inform us that *𝜃* is likely to be higher than 0.5 (see also Etz & Vandekerckhove, 2018, Example 5); indeed, the 95% credible interval ranges from 0.486 to 0.808.

The idea of Verhagen and Wagenmakers was to use this posterior from the first experiment as an informed prior for a second experiment. This is in accordance with Bayesian parameter updating and the adage “today’s posterior is tomorrow’s prior” (Lindley, 1972, p. 2). The resulting “replication Bayes factor” quantifies the relative predictive adequacy of the null hypothesis versus an alternative hypothesis that is completely informed by the knowledge of the parameter obtained from the first study.

To demonstrate the procedure, consider the second experiment conducted by Krupenye et al., (2016):

“In experiment two, we tested 30 subjects (29 from experiment one, plus one additional bonobo). Twenty-two apes made explicit looks to the target or the distractor during this period. Of these 22, 17 looked first at the target (*P* = 0.016, two-tailed binomial test)” (Krupenye et al., 2016, p. 113).

In order to compute the replication Bayes factor, we take the posterior distribution from Experiment 1 (i.e., the solid line in Fig. 1), and use it as a prior distribution for the analysis of the second experiment. Recall that the original uniform prior was a Beta(1,1) distribution; after incorporating the 20 successes and ten failures from the first experiment, the posterior remains a beta distribution, namely, Beta(1 + 20,1 + 10). This distribution can be specified in the Summary Stats module of JASP.

**Fig. 2:**
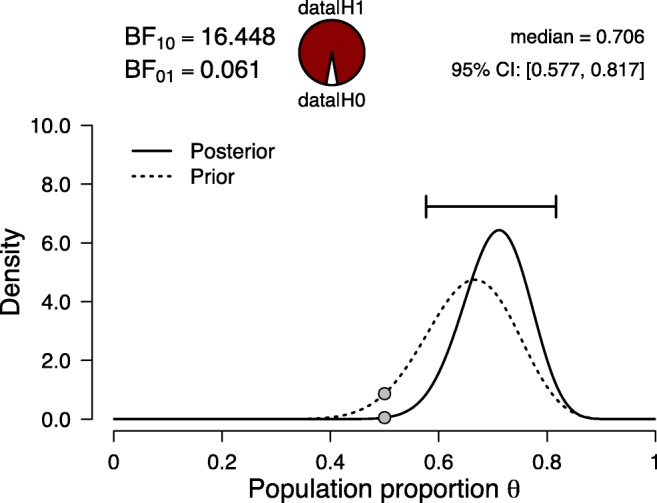
Bayesian reanalysis of the results from the second experiment in Krupenye et al., (2016)—where 17 out of 22 apes (≈ 77*%*) first looked at the target—after having updated *𝜃* using the data from the first experiment. Figure from JASP

The result is displayed in Fig. 2. The dashed line quantifies the knowledge of an idealized proponent, who believes the effect is present and has access to the data from Experiment 1. The solid line is the posterior distribution when this knowledge has been updated using the data from Experiment 2. This posterior distribution does not assign much mass to values of *𝜃* near 0.5, and consequently the replication Bayes factor is relatively strong: the data are about 16 times more likely under the proponent’s $\mathcal {H}_{r}$ than under the skeptic’s $\mathcal {H}_{0}$.

This process of updating to a posterior and then using it as a prior for the analysis of the next experiment is relatively straightforward for this simple example. For more complex models, however, the process can be burdensome, approximate, and intricate. In the remainder of this paper, we will propose an easier, more exact way forward that focuses on updating the evidence rather than the parameter priors.

## The replication Bayes factor reconceptualized

The example above demonstrated how the replication Bayes factor can be obtained by a standard Bayesian parameter updating process, that is, by using the posterior distribution from the first experiment as a prior distribution for the replication test of the second experiment.

**Fig. 3:**
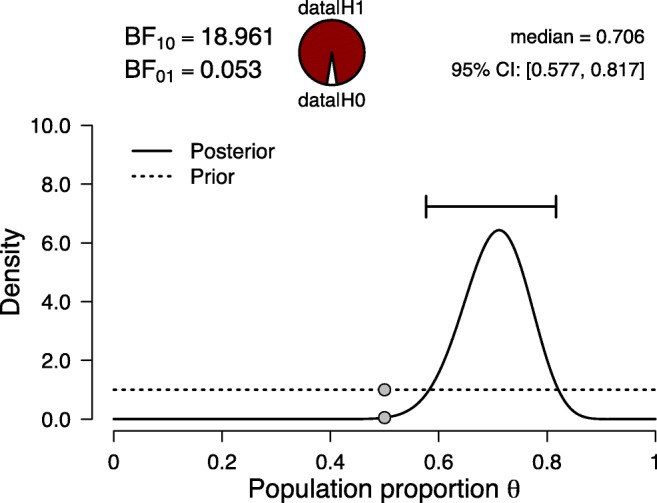
Bayesian reanalysis of the results from the first and second experiment in Krupenye et al., (2016) combined, where 37 out of 52 apes (≈ *%*71) first looked at the target. Figure from JASP

However, there exists a simpler way to obtain the replication Bayes factor, one that does not explicitly require the parameter updating process. To explain this alternative method, we revisit Krupenye et al., (2016) and analyze the data from both experiments together (i.e., 20 + 17 = 37 first looks at the target out of 30 + 22 = 52 trials). Figure 3 shows the results. The posterior distribution equals the one shown in Fig. 2; in other words, it does not matter whether the original prior distribution is updated in two steps—first the data from Experiment 1, then the data from Experiment 2—or all at once. Crucially, this property also holds for the Bayes factor (e.g., Jeffreys, 1938, pp. 190–192). The Bayes factor for the combined result, shown in Fig. 3, equals 18.961. The Bayes factor for the first experiment equals 1.153 (see Fig. 1), and the Bayes factor for the second experiment—after updating based on the knowledge obtained in the first experiment—equals 16.448 (see Fig. 2).^1^ Multiplying these two Bayes factors yields 1.153 × 16.448 = 18.965, the same result as is obtained when all data are analyzed at once.^2^

In other words, the multiplication of component Bayes factors, when properly updated, yields the complete Bayes factor:

4 $$ \underbrace{ \text{BF}_{10}(d_{\text{orig}}, d_{\text{rep}})}_{\text{Complete BF}} = \underbrace{ \text{BF}_{10}(d_{\text{orig}})}_{\begin{array}{cccccccc}\text{BF original}\\ \text{experiment} \end{array} } \times \underbrace{ \text{BF}_{10}(d_{\text{rep}} | d_{\text{orig}})}_{\text{Replication BF}}, $$

where *d*_orig_ denotes the data from the original study, and *d*_rep_ the data from the replication attempt. Note that the replication Bayes factor is the change in the Bayes factor due to the observation of the replication data, and quantifies the additional evidence for the alternative hypothesis given what was already observed in the original study.

Rearranging (4) then yields the crucial identity

5 $$ \text{BF}_{10}(d_{\text{rep}} | d_{\text{orig}}) = \frac{ \text{BF}_{10}(d_{\text{orig}}, d_{\text{rep}})}{ \text{BF}_{10}(d_{\text{orig}})}, $$

which shows that the replication Bayes factor may be obtained by dividing the complete Bayes factor by the Bayes factor from the original experiment. Importantly, the replication Bayes factor is obtained much easier by updating the evidence than by updating the parameters, as the evidence-updating procedure does not require the researcher to approximate the posterior from the original study and specify it in a software program. For complex models, this requirement is prohibitive. We now turn to additional examples that demonstrate the ease with which the evidence-updating (henceforth “EU”) replication Bayes factor can be obtained.

## Example 1: a *t* test to assess whether superstition improves performance

Consider perhaps the most routine replication scenario, one where a researcher conducts a replication of a study whose analysis featured a *t* test. For a common *t* test, JASP allows the specification of a Cauchy, *t*, or normal prior for the effect size *δ* and the user is free to specify the center and scale of this prior (for technical details see Gronau, Ly, & Wagenmakers, 2017a). However, in contrast to parameter *𝜃* from the binomial test, the posterior for *δ* in a *t* test has no known distributional form. The applied scientist is therefore unable to use the posterior as a prior to calculate a replication Bayes factor in JASP.

To overcome this hurdle, Verhagen and Wagenmakers (2014) proposed to approximate the posterior on effect size obtained from the *t* test with a normal distribution; this normal distribution is then used as a prior for the analysis of the replication experiment. Unfortunately, this approximation in the intermediate step between the original and the replication study makes this method computationally involved and hard to generalize to other designs.

To illustrate the simplicity of the EU replication Bayes factor, we revisit a recently published replication study by Calin-Jageman and Caldwell (2014) on the effect of superstition and performance in golf players (Damisch et al., 2010). The authors summarized the background as follows:

“Can superstitions actually improve performance? Damisch et al., (2010) reported a striking experiment in which manipulating superstitious feelings markedly increased golfing ability. Participants attempted ten putts, each from a distance of 100 cm. Some participants were primed for superstition prior to the task by being told ‘Here is the ball. So far it has turned out to be a lucky ball.’ Controls were simply told ‘This is the ball everyone has used so far.’ Remarkably, this manipulation produced a substantial increase in golf performance: Controls made 48*%* of putts while superstition-primed participants made 65*%* of putts (*d* = 0.83, 95*%* CI [0.05,1.60]).” (Calin-Jageman & Caldwell, 2014, p. 239)

**Fig. 4:**
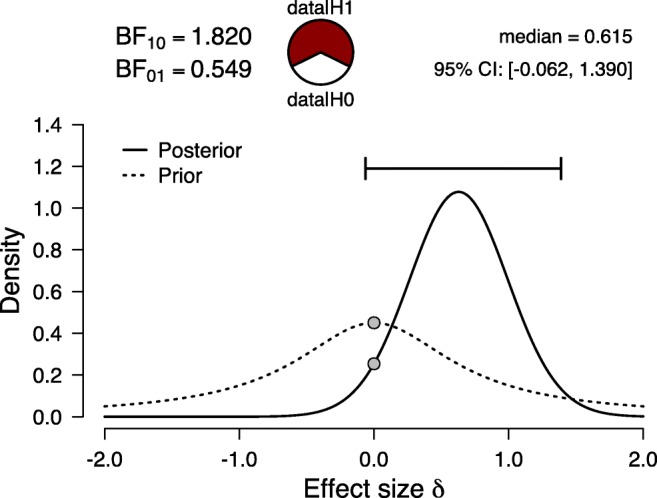
Bayesian reanalysis of the original results from Experiment 1 of Damisch et al., (2010), where golfers who played with a “lucky” ball made more putts (*t*(26) = 2.14,*p* = .042,*d* = 0.83). Figure from JASP

A classical *t* test^3^ of the original data resulted in a statistically significant result, *t*(26) = 2.14,*p* = .042,*d* = 0.83. As shown in Fig. 4, a Bayesian independent-samples *t* test using the JASP Summary Stats module returns BF_10_(*d*_orig_) = 1.820, a level of evidence that is not compelling. Calin-Jageman and Caldwell (2014) performed a direct replication of this work. Their Experiment 1 featured 58 control participants and 66 “superstition-activated” participants. The latter group outperformed the controls by only 2%, a result that is not statistically significant (i.e., *t*(122) = 0.29,*p* = .77,*d* = 0.05).

To compute the EU replication Bayes factor, we first need to compute the complete Bayes factor for these two data sets. Since both the original and replication papers report the raw means and standard deviations for each of the two groups (which are sufficient statistics for the *t* test, see Ly, Marsman, Verhagen, Grasman, & Wagenmakers, 2017), we can straightforwardly compute the overall *t* value for the combined data (see Appendix A for a description of the algebra involved); this yields an overall *t* = 1.14, which corresponds to a complete Bayes factor of BF_10_(*d*_orig_,*d*_rep_) = 0.318. The replication Bayes factor can now be obtained by simply dividing the complete Bayes factor by the Bayes factor from the original data alone and leads to BF_10_(*d*_rep_|*d*_orig_) = 0.175. In other words, the skeptic’s null hypothesis predicted the data from the replication attempt 1/0.175 = 5.72 times better than the proponent’s alternative hypothesis informed by the original data set.

## Example 2: a contingency table analysis to test whether more valuable stimuli are judged to be relatively rare

The previous example featured a *t* test and therefore the replication Bayes factor could also have been approximated using the parameter-updating procedure outlined in Verhagen and Wagenmakers (2014). We now turn to an example for which this parameter-updating procedure is problematic: the default Bayesian test for independence in a contingency table (Gunel & Dickey, 1974; Jamil et al., 2017).

The test for independence involves the construction of a model that is more complex than the models used for the *t* test. Consequently, in JASP, the researcher can only input a parameter that governs the relative concentration of the joint prior distribution, and—for the special case of a 2 × 2 table—receive a posterior distribution for the log-odds ratio, a derived summary measure that quantifies the degree of association. This generic setup does not allow researchers to obtain a joint parameter posterior from past studies and use it as a prior for current studies, frustrating the parameter-updating version of the replication Bayes factor.

However, a contingency table replication test is straightforwardly implemented by using the EU replication Bayes factor, as we now demonstrate by an example taken from the Reproducibility Project: Psychology (RP:P; Open Science Collaboration, 2015). As part of the RP:P, Fuchs, Estel, and Göllner performed a replication of a study by Dai et al., (2008), who

“(…) tested a novel heuristic for making judgments of relative frequency. According to this so-called value heuristic, ‘people judge the frequency of a class of objects on the basis of the subjective value of the objects’ (p. 18). Based on the principle that scarcity increases an object’s value, the authors [Dai et al.] formulate the hypothesis that individuals will assess more valuable stimulus classes to be less frequent even when value is not diagnostic of frequency.”

The data from Dai and colleagues’ original study are presented in Table 1. The raw data suggest that endowing a category leads participants to judge that category as having fewer occurrences, in line with their original hypothesis. Subjecting this original finding to a classical contingency table test results in *χ*^2^(1,56) = 4.51, *p* = .037, and a default Bayesian reanalysis (Gunel & Dickey, 1974) using JASP yields BF_10_(*d*_orig_) = 2.880.

**Table 1:** Data from Dai et al., (2008), who concluded that endowing a category may lead participants to judge that category to be relatively rare

Endowed	Estimates		
	Fewer flowers	Fewer birds	Total
Flowers	15	12	27
Birds	8	21	29
Total	23	33	56

The data from Fuchs and colleagues’ replication attempt are shown in Table 2. A classical contingency table test applied to these data returns *χ*^2^(1,51) = 1.57, *p* = .21, which is not statistically significant. To reanalyze this data using our EU replication Bayes factor, we first combine the data into a single sample (see Table 3) and compute the complete Bayes factor, BF_10_(*d*_orig_,*d*_rep_) = 0.298. To obtain the replication Bayes factor, we simply divide BF_10_(*d*_orig_,*d*_rep_) by BF_10_(*d*_orig_), which yields BF_10_(*d*_rep_|*d*_orig_) = 0.103. This means that the replication data are predicted 1/0.103 = 9.71 times better by the null hypothesis than by the alternative hypothesis informed by the original data set.^4^

**Table 2:** Data from the replication experiment by Fuchs and colleagues

Endowed	Estimates		
	Fewer flowers	Fewer birds	Total
Flowers	11	16	27
Birds	14	10	24
Total	25	26	51

The data do not support the original finding of Dai et al., (2008)

**Table 3:** Data from the original and replication experiment combined

Endowed	Estimates		
	Fewer flowers	Fewer birds	Total
Flowers	26	28	54
Birds	22	31	53
Total	48	59	107

Note that this pooling procedure assumes that the data are exchangeable, that is, it presumes that the replication study is direct and close

## Conclusions

The replication Bayes factor (Verhagen & Wagenmakers, 2014) provides an intuitive measure of replication success: rather than ignoring the original study, the replication Bayes factor uses the posterior distribution obtained from the original study as a prior distribution for the test of the data from the replication study.

Here we provided an additional perspective on the replication Bayes factor, namely as the change in evidence brought about by observing the results from the replication study. The advantage of this “evidence-updating” or EU perspective on the replication Bayes factor is that it does not require approximations, and that it can be easily applied to complex models. One reviewer noted that the EU replication Bayes factor follows directly from the general properties of the Bayes factor. Although this assessment is correct, we nevertheless believe that the EU replication Bayes factor represents a conceptual and practical advance. As is often the case in probability theory, solutions appear trivial only after they have been derived. In this particular case, Verhagen and Wagenmakers (2014) were unaware of the EU replication Bayes factor; in general, it is not immediately obvious that the parameter updating step—an integral part of the original Verhagen and Wagenmakers method—can be entirely omitted.

Both the original parameter-updating version and the current EU version of the replication Bayes factor are based on the idea of evidence synthesis and scientific learning (e.g., Marsman, Ly, & Wagenmakers, 2016; Scheibehenne et al., 2016; Silber et al., 2016). With more than two studies, the proposed method is similar to a fixed-effects meta-analysis that assumes the data to be exchangeable.^5^

As with any statistical method, it can become vulnerable when its core assumptions are violated. For the EU replication Bayes factor, the most serious threat to its validity arises when the replication is not close, and aspects differ that the model assumes to be the same. Consider the *t* test. The parameter-updating version updates only the test-relevant parameter *δ*, but the nuisance parameters (e.g., the grand mean, which is common to $\mathcal {H}_{0}$ and $\mathcal {H}_{1}$), were not updated. This small omission is rectified by the EU version that automatically and implicitly updates the joint prior for all model parameters. However, this updating of nuisance parameters also creates a lack of robustness: when the nuisance parameters do undergo a large change from original to replication study, the results can be misleading. For instance, assume that a replication attempt successfully reproduces the main effect of condition, but all participants are 150 ms slower. When the raw data from the two studies are combined, this artificially inflates the variance and may make it appear as if the replication failed.

A similar warning applies for a correlation test, where the parameter of interest—the correlation coefficient *ρ*—may be of similar magnitude in the original and the replication study, but global changes in the location parameters of the bivariate normal distribution can skew the outcome of the EU replication Bayes factor. For instance, suppose one studies the relation between income and body weight. The replication attempt finds the same correlation but on average participants make $10,000 more and weigh 15 pounds less. Visually, this yields two clouds of points; each may have the same shape and orientation, but pooling the raw data may create a misleading impression.

The solution to this lack of robustness is two-fold. First, users must be aware that this is a potential problem. Second, the data may be transformed to absorb any changes in nuisance parameters. For instance, correlational data may be mean-centered before being combined.

Another vulnerability of the replication Bayes factor (regardless of whether it is the parameter-updating version or the EU version) is that, in rare cases, it brings about a replication paradox. The paradox is that when a replication attempt strongly suggests that the results go in the direction opposite to the one found in the original study, the replication Bayes factor may yield compelling evidence in favor of the alternative hypothesis that the effect has successfully replicated. As with all uses of probability theory, such paradoxes reveal a lack of proper understanding. Appendix C illustrates the paradox and explains that it can be resolved by imposing an order restriction.

No single measure of replication success suffices to address all questions that surround the interpretation of a replication attempt. We advocate an inclusive approach to the statistical assessment of replication success, and we hope that the EU replication Bayes factor can be one of many tools that are at researchers’ disposal, to be applied not just across laboratories but also within laboratories.

## Figures and Tables

**Fig. 5:**
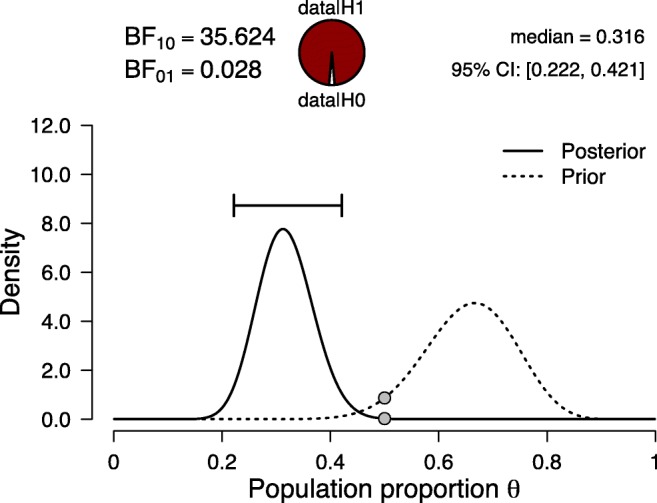
A replication paradox. In the first experiment by Krupenye et al., (2016), 20 out of 30 apes (i.e., ≈ 67*%*) had looked at the target first; in a hypothetical replication experiment, only five out of 50 apes did so (i.e., 10%). The effect in the hypothetical replication attempt goes in the direction opposite to that of the original study, and yet the replication Bayes factor indicates strong support in favor of the proponent’s alternative hypothesis. Figure from JASP

**Fig. 6:**
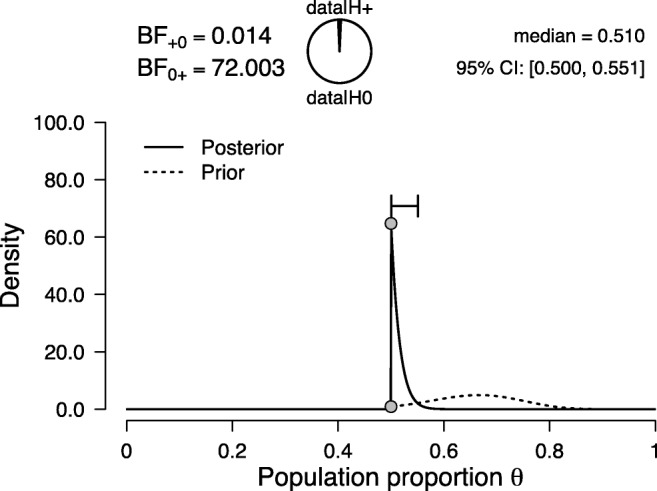
A replication paradox resolved. In the first experiment by Krupenye et al., (2016), 20 out of 30 apes (i.e., ≈ 67*%*) had looked at the target first; in a hypothetical replication experiment, only five out of 50 apes did so (i.e., 10%). The effect in the hypothetical replication attempt goes in the direction opposite to that of the original study. By imposing an order restriction and allowing *𝜃* to take on only values larger than 0.5, the replication Bayes factor now indicates strong support in favor of the skeptic’s null hypothesis. Figure from JASP

## Appendix A: Deriving the *t* value across both data sets

The two-sample *t*-statistic over the combined data *d*_all_ = (*d*_orig_,*d*_rep_) can be computed from the sample means and variances of the two data sets

6 $$\begin{array}{@{}rcl@{}} d_{\text{orig}} & =& (n_{\text{orig}, x}, \bar{x}_{\text{orig}}, s^{2}_{\text{orig}, x}, n_{\text{orig}, y}, \bar{y}_{\text{orig}}, s^{2}_{\text{orig}, y}), \end{array} $$

7 $$\begin{array}{@{}rcl@{}} d_{\text{rep}} & =& (n_{\text{rep}, x}, \bar{x}_{\text{rep}}, s^{2}_{\text{rep},x}, n_{\text{rep}, y}, \bar{y}_{\text{rep}}, s^{2}_{\text{rep}, y}), \end{array} $$

where *n*_orig,*x*_,*n*_orig,*y*_ are the sample sizes, *x̄*_orig_,*ȳ*_orig_ are the sample means, and *s̄* orig,*x*2,*s̄* orig,*y*2 the (unbiased) sample variances of the first (i.e., “*x*”) and second group (i.e., “*y*”) from the original data set. The same symbols with orig replaced by rep have an analogous meaning. The combined two-sample *t*-statistic under the assumption of equal population variance is then given by

8 $$ t_{\text{all}} = \frac{\bar{x}_{\text{all}} - \bar{y}_{\text{all}}}{\sqrt{s^{2} [\frac{1}{n_{\text{all}, x}} + \frac{1}{n_{\text{all}, y}}]}}, $$

where *n*_all,*x*_ = *n*_orig,*x*_ + *n*_rep,*x*_ and *n*_all,*y*_ = *n*_orig,*y*_ + *n*_rep,*y*_ are the combined sample sizes of the first and second group respectively, and where

9 $$\begin{array}{@{}rcl@{}} \bar{x}_{\text{all}} & =& \frac{n_{\text{orig}, x} \bar{x}_{\text{orig}} + n_{\text{rep}, x} \bar{x}_{\text{rep}}}{n_{\text{all}, x}}, \end{array} $$

10 $$\begin{array}{@{}rcl@{}} \bar{y}_{\text{all}} & =& \frac{ n_{\text{orig}, y} \bar{y}_{\text{orig}} + n_{\text{rep}, y} \bar{y}_{\text{rep}}}{n_{\text{all}, y}}, \end{array} $$

are the combined means of the two groups and

11 $$ s^{2} = \frac{1}{n_{\text{all}, x} + n_{\text{all}, y} -2 } \left[\sum\limits_{i = 1}^{n_{\text{all}, x}} (x_{i} - \bar{x}_{\text{all}})^{2} + \sum\limits_{i = 1}^{n_{\text{all}, y}} (y_{i} - \bar{y}_{\text{all}})^{2} \right] , $$

the combined (pooled) sample variance, where

12 $$\begin{array}{@{}rcl@{}} \sum\limits_{i = 1}^{n_{\text{all}, x}} (x_{i} - \bar{x}_{\text{all}})^{2} & = &\nu_{\text{orig}, x} s_{\text{orig}, x}^{2} + n_{\text{orig}, x} \bar{x}^{2}_{\text{orig}} \\&&+\nu_{\text{rep}, x} s_{\text{rep}, x}^{2} + n_{\text{rep}, x} \bar{x}^{2}_{\text{rep}} - n_{\text{all}, x} \bar{x}^{2}_{\text{all}} \end{array} $$

13 $$\begin{array}{@{}rcl@{}} \sum\limits_{i = 1}^{n_{\text{all}, y}} (y_{i} - \bar{y}_{\text{all}})^{2} & = & \nu_{\text{orig}, y} s_{\text{orig}, y}^{2} + n_{\text{orig}, y} \bar{y}^{2}_{\text{orig}} \\&&+ \nu_{\text{rep}, y} s_{\text{rep}, y}^{2} + n_{\text{rep}, y} \bar{y}^{2}_{\text{rep}} - n_{\text{all}, y} \bar{y}^{2}_{\text{all}} \end{array} $$

are the combined sums of squares of the first and second group, respectively, with *ν*_orig,*x*_ = *n*_orig,*x*_ − 1 and *ν*_orig,*y*_ = *n*_orig,*y*_ − 1 denoting the degrees of freedom.

### Proof

The combined mean of the first group follows from the equality

14 $$ n_{\text{all}} \bar{x}_{\text{all}} = \sum\limits_{i = 1}^{n_{\text{all}, x}} x_{i} = n_{\text{orig}, x} \bar{x}_{\text{orig}} + n_{\text{rep}, x} \bar{x}_{\text{rep}}, $$

and the combined mean of the second group can be derived analogously. Recall that the sums of squares ∑ (*x*_*i*_ −*x̄*)^2^ equals the sum of the squares centered at zero minus *n* times the square of the mean, that is,

15 $$ \nu_{\text{orig}, x} s_{\text{orig}, x}^{2} = \sum\limits_{i = 1}^{n_{\text{orig}, x}} (x_{\text{orig}, 1} - \bar{x}_{\text{orig}})^{2} = \left( \sum\limits_{i = 1}^{n_{\text{orig}, x}} x_{\text{orig}, i}^{2} \right) - n_{\text{orig}, x} \bar{x}_{\text{orig}}^{2} . $$

The same holds for the sums of squares of the replication data and the combined data *d*_all_. As such, we can write the first sums of squares in the numerator of *s*^2^ as

16 $$\begin{array}{@{}rcl@{}} \sum\limits_{i = 1}^{n_{\text{all}, x}} (x_{i} - \bar{x}_{\text{all}})^{2} & = & \nu_{\text{orig}, x} s_{\text{orig}, x}^{2} + n_{\text{orig}, x} \bar{x}^{2}_{\text{orig}} \\&&+\nu_{\text{rep}, x} s_{\text{rep}, x}^{2} + n_{\text{rep}, x} \bar{x}^{2}_{\text{rep}} - n_{\text{all}, x} \bar{x}^{2}_{\text{all}} \end{array} $$

and the derivation is similar for *y*. □

## Appendix B: Replication Bayes factors as conditional Bayes factors

Let *d*_orig_,*d*_rep_ be exchangeable and write *π*(*𝜃*_0_|*d*_orig_) and *π*(*𝜃*_1_|*d*_orig_) for the posterior for the parameters of the null model and alternative model respectively. Thus,

17 $$ \pi(\theta_{j} | d_{\text{orig}}) = \frac{ f(d_{\text{orig}} | \theta_{j}) \pi(\theta_{j}) }{ \int f(d_{\text{orig}} | \theta_{j}) \pi(\theta_{j}) \mathrm{d} \theta_{j}} = \frac{ f(d_{\text{orig}} | \theta_{j}) \pi(\theta_{j}) }{ p(d_{\text{orig}} | \mathcal{H}_{j}) } $$

where $p(d_{\text {orig}} | \mathcal {H}_{j})$ is the marginal likelihood of hypothesis $\mathcal {H}_{j}$. The procedure that uses the posterior based on the original data set *d*_orig_ as a prior for the replication data set can now be rewritten as

18 $$\begin{array}{@{}rcl@{}} \text{BF}_{r0}(d_{\text{rep}}) & = & \frac{\int f(d_{\text{rep}} | \theta_{1}) \pi(\theta_{1} | d_{\text{orig}}) \mathrm{d} \theta_{1}}{ \int f(d_{\text{rep}} | \theta_{0}) \pi(\theta_{0} | d_{\text{orig}}) \mathrm{d} \theta_{0} }, \end{array} $$

19 $$\begin{array}{@{}rcl@{}} & = & \frac{p(d_{\text{orig}} | \mathcal{H}_{0})}{p(d_{\text{orig}} | \mathcal{H}_{1})}\frac{\int f(d_{\text{rep}} | \theta_{1}) f(d_{\text{orig}} | \theta_{1}) \pi (\theta_{1}) \mathrm{d} \theta_{1}} { \int f(d_{\text{rep}} | \theta_{0}) f(d_{\text{orig}} | \theta_{0}) \pi(\theta_{0} ) \mathrm{d} \theta_{0} }, \end{array} $$

20 $$\begin{array}{@{}rcl@{}} & = & \text{BF}_{01}(d_{\text{orig}}) \text{BF}_{10}(d_{\text{orig}}, d_{\text{rep}}) = \frac{\text{BF}_{10}(d_{\text{orig}}, d_{\text{rep}})}{\text{BF}_{10}(d_{\text{orig}})}, \end{array} $$

21 $$\begin{array}{@{}rcl@{}} & = & \text{BF}_{10}(d_{\text{orig}} | d_{\text{rep}}). \end{array} $$

Hence, the parameter-updating and evidence-updating replication Bayes factor are equivalent to each other under the assumption that *d*_orig_ and *d*_rep_ are exchangeable and the fixed effect assumption.

## Appendix C: Replication paradox and solution

Regardless of whether it is calculated from parameter-updating or evidence-updating, the replication Bayes factor can produce a paradoxical result whenever the data from a replication attempt strongly indicate that the result is in the direction opposite of the one obtained in the original experiment. Here we illustrate the paradox and explain its resolution.

For concreteness, assume that the original experiment is the first study of Krupenye et al., (2016), where 20 out of 30 apes first looked at the target (see Fig. 1). Now imagine a hypothetical replication in which only five out of 50 apes look at the target, contradicting the direction of the original effect. One may intuit that this disappointing result indicates compelling evidence against the proponent’s alternative hypothesis as given by the posterior distribution from Fig. 1. Surprisingly, however, Fig. 5 indicates that the Bayes factor is 35.6 in favor of the proponent’s alternative hypothesis.

The key insight is to realize that the replication Bayes factor—just as other Bayes factors—quantifies relative evidence. With only five out of 50 looks at the target, the null hypothesis utterly fails to account for the data. The proponent’s $\mathcal {H}_{r}$ as specified by the dashed line in Fig. 5 also predicts these data poorly but not across all of its parameter space; indeed, $\mathcal {H}_{r}$ has some prior mass on values of *𝜃* below 0.5. This resolves the paradox. The surprise at the support for the proponent’s hypothesis (when the replication results contra-indicate the direction found in the original study) reflects the implicit notion that the proponent’s hypothesis ought to have a direction. Specifically, in the Krupenye et al., (2016) example, the authors clearly had a direction in mind when they discussed their findings. Consider the same test but now impose the restriction that *𝜃* ≥ 0.5. The result is shown in Fig. 6; now the Bayes factor is 72 in favor of the null hypothesis.

Generally, we advocate the use of order restrictions to create more informative tests of the underlying theory (e.g., Matzke et al., 2015). However, it should be kept in mind that such order restrictions blind the researcher to the possibility that the effect might actually go in the direction opposite to that postulated by theory. When the data suggest that this may indeed be the case, follow-up experiments may instantiate this novel prediction as a new hypothesis and examine its adequacy.

## Footnotes

1 For a warning concerning the multiplication of Bayes factors that have not been properly updated, see Jeffreys (1938, pp. 190–192, 1961, Section 6.0), and Wagenmakers et al., (2015).

2 The difference between 18.965 and 18.961 is due to rounding and vanishes as the number of decimal places in the calculation are increased. The number of decimal places that are displayed in JASP can be increased in the preference window.

3 This analysis is consistent with the one used in the original experiment and the replication attempt. A more appropriate statistical analysis arguably uses a hierarchical binomial model.

4 It is worth noting that the replication Bayes factor in this example can be well approximated by a Bayes factor based on a normal prior and a normal likelihood (possibly after a suitable transformation of the parameters and the data; see Dienes & Mclatchie, 2018), as was brought to our attention by a reviewer. The normal prior is used as an approximation of the posterior (as in Verhagen & Wagenmakers, 2014), and the normal likelihood is used as an approximation to the exact likelihood. Specifically, the reviewer approximated the likelihood by a normal distribution with a mean of − 0.711 (i.e., the logarithm of the observed odds ratio in the replication study) and a standard deviation of 0.5699, and used as prior a normal distribution based on the logarithm of the odds ratio of 1.188, and a standard error of 0.568 as was observed from the original study. The reviewer then used the calculator proposed by Dienes (2008, 2014) and Dienes et al., (2018), which resulted in a Bayes factor of 0.10. See van Doorn et al., (2016) for a similar use of approximating normal likelihood to compute Bayes factors, and Ly et al., (2017) for some theoretical background.

5 For an extension of the methodology to random-effects models and model-averaging, see Gronau et al., (2017b) and Scheibehenne et al., (2017).
